# Thin and long silver nanowires self-assembled in ionic liquids as a soft template: electrical and optical properties

**DOI:** 10.1186/1556-276X-9-330

**Published:** 2014-07-03

**Authors:** Min-Hwa Chang, Hyun-Ah Cho, Youn-Soo Kim, Eun-Jong Lee, Jin-Yeol Kim

**Affiliations:** 1 School of Advanced Materials Engineering, Kookmin University, Seoul 136-702, South Korea

**Keywords:** Silver nanowire, Ionic liquid, Polyol synthesis, Tetrapropylammonium chloride, Tetrapropylammonium bromide, Self-assembly

## Abstract

Thin and long silver nanowires were successfully synthesized using the polyvinylpyrrolidone (PVP)-assisted polyol method in the presence of ionic liquids, tetrapropylammonium chloride and tetrapropylammonium bromide, which served as soft template salts. The first step involved the formation of Ag nanoparticles with a diameter of 40 to 50 nm through the reduction of silver nitrate. At the growing stage, the Ag nanoparticles were converted into thin and long one-dimensional wires, with uniform diameters of 30 ± 3 nm and lengths of up to 50 μm. These Ag nanowires showed an electrical conductivity of 0.3 × 10^5^ S/cm, while the sheet resistance of a two-dimensional percolating Ag nanowire network exhibited a value of 20 Ω/sq with an optical transmittance of 93% and a low haze value.

## Background

One-dimensional (1-D) metallic nanostructures, namely silver nanowires (Ag NWs), have recently attracted a great deal of attention for their unique electrical, optical, magnetic, and thermal properties as a promising alternative to indium tin oxide (ITO) as an electrode material used in the fabrication of devices such as electronic displays, photonics, and sensors [1-10]. Ag NWs with well-defined shapes such as lengths and diameters are particularly interesting, as they have superior optical and electrical properties, thus making them excellent candidates for transparent electrodes. However, in order to implement the optical and electrical features required for transparent electrodes, there is still a need to develop more effective processes for synthesizing Ag NWs with controllable shapes and sizes, which can be grown continuously up to at least 30 μm in length with 30-nm diameter. Several chemical approaches have been actively explored and developed in order to process Ag into 1-D nanostructures using various physical templates and surface-capping reagents (organic polymers or surfactants) in conjunction with the solution-phase polyol process [11-14]. These studies largely focused on controlling the size, shape, crystal structure, and optical/electrical properties of the Ag NWs. For example, Sun and co-workers [12] developed a solution-based polyol process to prepare single-crystal Ag NWs using polyvinylpyrrolidone (PVP) as a surface-capping reagent. The capping reagents were then evaluated in order to kinetically control the growth rates of the metal surfaces and subsequently induce 1-D growth leading to the formation of NWs. Based on the PVP-assisted polyol method, Xia and co-workers [15,16] also demonstrated a salt-mediated polyol process, using NaCl, CuCl_2_, PtCl_2_, or CuCl, to prepare Ag NWs of 30 to 60 nm in diameter in large quantities. Murphy et al. [17] first reported the preparation of Ag NWs with uniform diameters using the seed-mediated growth approach with a rodlike micelle template, cetyltrimethylammonium bromide (CTA-B), as the capping reagent. First, Ag seed nanoparticles with an average diameter of 4 nm were prepared by reducing AgNO_3_ with NaBH_4_ in the presence of trisodium citrate. The Ag seed particles were then grown into 1-D structures with a twinned crystal arrangement in the presence of the CTA-B capping reagent. Here, the capping reagent regulates this process by confining the growth of the lateral surface and including the expansion of the surface of the wire, leading to the formation of wires with a high aspect ratio. However, continuous Ag NWs of up to 40 μm in length with a small diameter of 30 nm have yet been synthesized via the polyol method.

**Figure 1:**
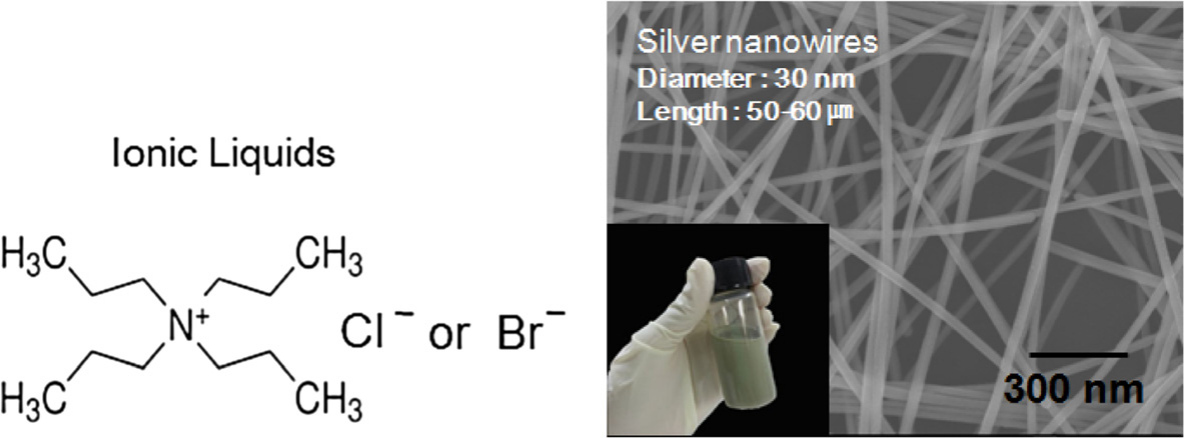
**Molecular structure of ILs and SEM image of Ag NWs.** Molecular structure of ILs composed of ammonium salts (TPA-C and TPA-B) (left) and the SEM image of Ag NWs synthesized in the presence of the ionic liquid (the inset shows a Ag NW sample solution dispersed in H_2_O) (right).

**Figure 2:**
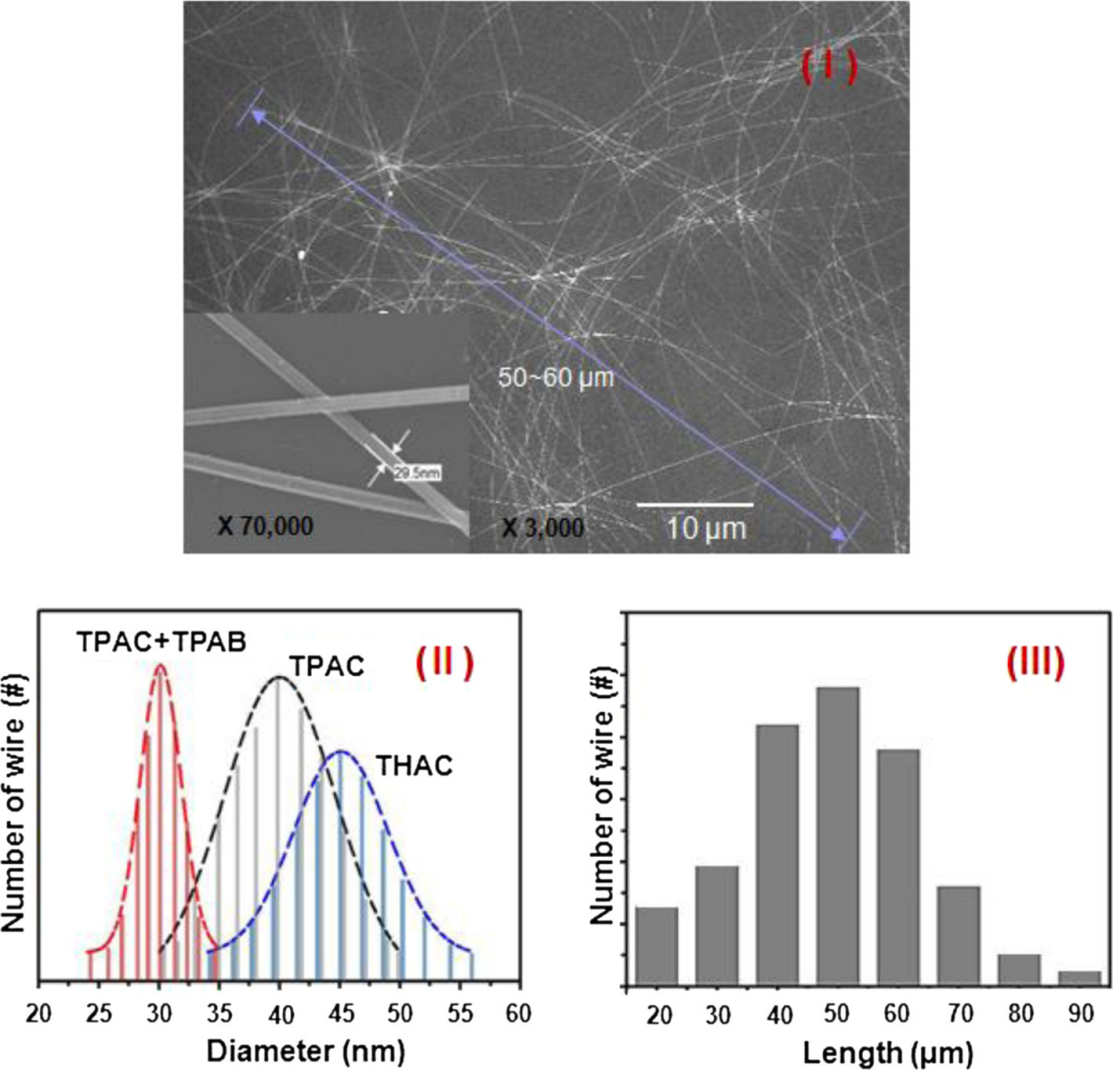
**SEM image and distributions of the diameter and the length of Ag NWs. (I)** SEM image of the Ag NWs synthesized using ionic liquid as a soft template. The inset is a large-scale SEM image of Ag NWs of approximately 30 nm in diameter. **(II)** Distributions of the diameter of the Ag NWs synthesized using various ILs (mixture of TPA-C and TPA-B, TPA-C, and THA-C). **(III)** Distributions of the length of the Ag NWs.

In this report, we demonstrate a new approach based on the PVP-assisted polyol method for the preparation of Ag NWs with a thin diameter (30 nm) and long length (40 to 60 μm) using ionic liquids (ILs), a mixture of tetrapropylammonium chloride (TPA-C) and tetrapropylammonium bromide (TPA-B), as soft template salts. TPA-C and TPA-B (Figure 1) are both classified as ILs, which are typically organic salts composed of organic cations of ammonium^+^ and anions of Cl^-^ and Br^-^. The properties of these liquids include extremely low volatilities, high thermal stabilities, a wide temperature range of the liquid phase, and high ionic conductivity [18-20]. A key feature of ILs is that their cations, anions, and substituents can be altered virtually at will in order to adjust their chemical and physical properties. In particular, the self-assembled local structures of ILs can effectively serve as templates for highly organized nanostructures. Additionally, the structure of the ILs associated with specific anions is known to self-organize in such a way that it is compliant to the fabrication of metal nanostructures [21]. In this regard, recently, Suh et al. [22] demonstrated that imidazolium salts as a kind of IL can be used as a reaction mediator capable of promoting the growth of Ag NWs, although the length of wires is short. Additionally, we also demonstrated that the self-assembled local structures of the imidazolium-based ILs can effectively serve as templates for highly organized nanostructures [23]. In this work, we examined that specific self-assembled local structures, and pores, may exist in an ammonium-based IL, thus demonstrating that ammonium IL can be effectively used as a soft template material capable of promoting the growth of Ag NWs. The IL-assisted formation of Ag NWs was performed, in which a metal precursor (AgNO_3_) was converted to elemental metal by ethylene glycol (EG) in the presence of ammonium ILs. The IL (which was composed of TPA-C and TPA-B) was then evaluated as a soft template in order to control the Ag nanostructures. During the initial step, Ag particles with a diameter of 40 to 50 nm were formed through the reduction of AgNO_3_ in the presence of ammonium ILs with the PVP capping reagent in EG. The Ag nanoparticles were then directly grown into 1-D single-crystal twinned structures, with uniform diameters in the range of 27 to 33 nm with long lengths of 40 to 60 μm (see Figure 1), in the PVP-based polyol conditions with TPA-C and TPA-B as the template as shown in Figure 1. In particular, the diameter of NWs was largely influenced by the type or pore size of IL, and their sizes could also be effectively and easily adjusted within a diameter range of 20 to 50 nm according to the ILs (see Figure 2). As the results show, this approach produces Ag NWs in high yields, making it very useful for the large-scale production of long and thin but uniform Ag NWs.

## Methods

Thin and uniform Ag NWs were synthesized through the chemical reduction of AgNO_3_ (Aldrich, St. Louis, MO, USA) with PVP (average molecular weight, *M*_w_ = 1,200,000) as a capping agent in the presence of a solution containing TPA-C and TPA-B. Approximately 35 mL (0.35 M in EG) of PVP, 15 mL (0.006 M in EG) of TPA-C, and 15 mL (0.003 M in EG) of TPA-B were simultaneously added to 170 mL of EG while being stirred at 120°C. Seventy milliliters (0.1 M in EG) of AgNO_3_ dissolved in 70 mL of EG was then added to the reaction mixture and stirred for 40 min. The reaction was carried out within an autoclave reactor. The reaction mixture was heated at 170°C for an additional 30 min during the wire growth stage. The final products, Ag NWs, were washed with acetone several times to remove the solvent (EG), PVP, and other impurities. After washing, the precipitate was re-dispersed in H_2_O.

The morphology and molecular structures of the resulting dispersed Ag NWs were observed by field emission scanning electron microscopy (FE-SEM; JEOL JSM-5410, Tokyo, Japan) and transmission electron microscopy (TEM; JEOL JEM-2100 F). The optical and surface plasmon resonance (SPR) spectra were measured using ultraviolet spectroscopy (UV/vis, SHIMADZU UV-3150, Tokyo, Japan). Conductivity was measured using the standard four-point probe technique.

## Results and discussion

By utilizing the experimental method mentioned above, we fabricated self-organized Ag NWs by reducing AgNO_3_ within the micelles of TPA salt templates, which are ammonium-based IL. This did not need any additional ions required to control the crystal growth of silvers and utilized PVP as the surface capping reagent. Surprisingly, during the first synthetic step in the building of the Ag nanostructures, Ag nanoparticles with a diameter of approximately tens of nanometers were found to exist and were subsequently converted into well-defined long wire structures. In this procedure, the diameter of the Ag nanoparticles and the Ag NWs is largely dependent on the type and amount of the ILs present in the reaction mixture. For example, the diameters of the Ag NWs produced from IL solutions of TPA-C and TPA-B mixture, TPA-C, and tetrahexylammonium chloride (THA-C) were 25 to 35 nm, 30 to 50 nm, and 35 to 55 nm, respectively, and their dispersions were also relatively wide, as shown in Figure 2II. These results confirm that there is a correlation between the sizes of the pore, micelle, and ILs employed as the soft template. In order to obtain finer and more uniform nanostructures, TPA-C was mixed with TPA-B in a ratio of 2:1 and subsequently utilized as soft template salts. The Ag nanostructures then formed Ag nanoparticles with a diameter of 30 to 40 nm during the initial reaction step and were subsequently converted into well-defined Ag NWs with a narrow and uniform diameter dispersion in the range of 27 to 33 nm and long length of up to 50 μm, as shown in Figure 2. Figure 2I displays an SEM image of the thin and long Ag NWs synthesized using the TPA-C and TPA-B mixture, while Figure 2II,III displays the distributions of the diameter and length, respectively, of the synthesized wires. Therefore, we determined that the diameter of the wires was affected more significantly than the length of the wire when the type and components of the ILs were varied. Then, the IL solutions appear to act as a size-controllable template salt within the liquid phase. In particular, the diameters of the Ag NWs were influenced by the type and components of the ILs, and their sizes could be effectively controlled within a diameter range of 20 to 50 nm according to the components of ILs.

**Figure 3:**
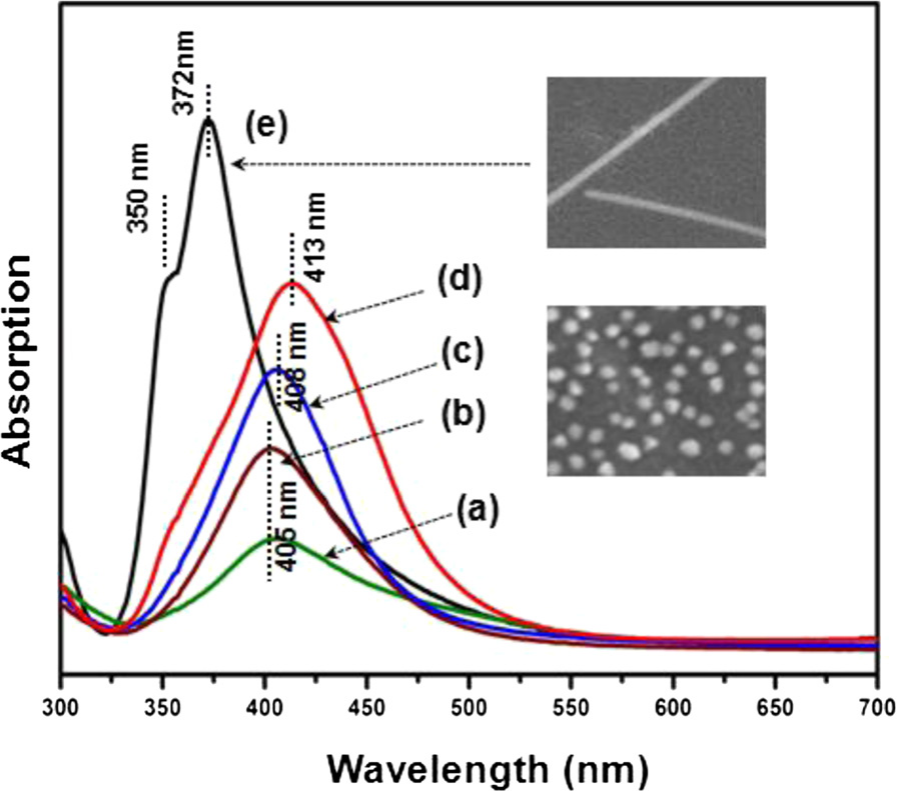
**SPR spectra measured every 10 min throughout the Ag NW synthesis.** SPR spectra obtained from the reaction after (a) 5 min, (b) 15 min, (c) 25 min, (d) 35 min, and (e) 60 min (inset figures: the Ag nanostructures, at the initial reaction step, existed as Ag particles of 40 to 50 nm in diameter, and after 60 min, these Ag particles were converted into a 1-D structure approximately 30 nm in diameter).

In order to identify the growth process, surface plasmon resonance (SPR) was observed at each stage of the synthesis reaction. It has been well documented that nanosized metals, especially Ag nanostructures, exhibit a wide range of optical phenomena directly related to SPR, depending on the geometry and size of the metal particles [24,25]. To demonstrate the specific ways in which the shape of silver wires affects the absorption and scattering of light, UV/vis spectroscopy was employed, analyzing the same materials used for electron microscopy. In general, a SPR spectrum can be fundamentally used to determine the size and shape of the Ag NW by examining the different SPR bands that appear at different frequencies. In this work, the growth process of Ag nanostructures was also studied by observing the SPR spectra. In order to monitor the growth process of the NWs, the SPR spectrum of the samples was measured, and the SPR peaks were determined every 10 min as shown in Figure 3. According to previous reports [26,27], the characteristic main SPR peaks for Ag NWs with diameter of 40 to 60 nm appear at approximately 350 and 380 nm. These peaks were attributed to the transversal modes of the 1-D product with pentagonal cross sections, which correspond to the out-of-plane quadrupole resonance and out-of-plane dipole resonance modes. The SPR bands of the Ag crystals (nanoparticles) with an edge length of 70 to 80 nm were also observed at 470 to 520 nm, as the peaks described above mutually overlap when mixtures containing Ag nanostructures of various shapes and sizes are analyzed. However, in this procedure, the formation of the Ag NWs was monitored by analyzing the SPR bands of the reaction mixture at various times (5, 15, 25, 35, and 60 min). The SPR peaks (Figure 3) can then be used to understand the mechanism of nanostructure growth. At the early stages of the reaction (10 min), the SPR band of the Ag nanoparticles with a size in the range of 30 to 40 nm formed through the reduction of AgNO_3_ in the presence of TPA exhibited a wavelength of 405 nm (Figure 3(a)). After a reaction of 40 min (Figure 3(d)), an absorption band appeared at 413 nm. On the other hand, Ag nanoparticles with an edge length of approximately 40 to 50 nm contained some multiply twinned crystals. As the reaction time increased (around 50 min), the Ag crystals were converted to pentagonal 1-D structures, while the Ag nanoparticles completely disappeared. At that time, as shown in Figure 3(e), the SPR absorption band clearly changed to the characteristic two peaks at 350 and 372 nm, which are indicative of wire formation. It is important to note that these two SPR peaks appear at significantly shorter wavelengths than the SPR peaks (350 and 380 nm) of the previously synthesized wires with diameters between 40 and 60 nm [26,27]. As a result, the blueshift originating from a reduction in the diameter of the NWs is also related to the reduction of scattered light. In addition to the blueshift phenomenon, a narrowing of the peak width was observed upon decreasing the NW diameter. However, ILs were also an important contributor in this assembly process as TPA supports the 1-D growth of the Ag nanoparticles.

**Figure 4:**
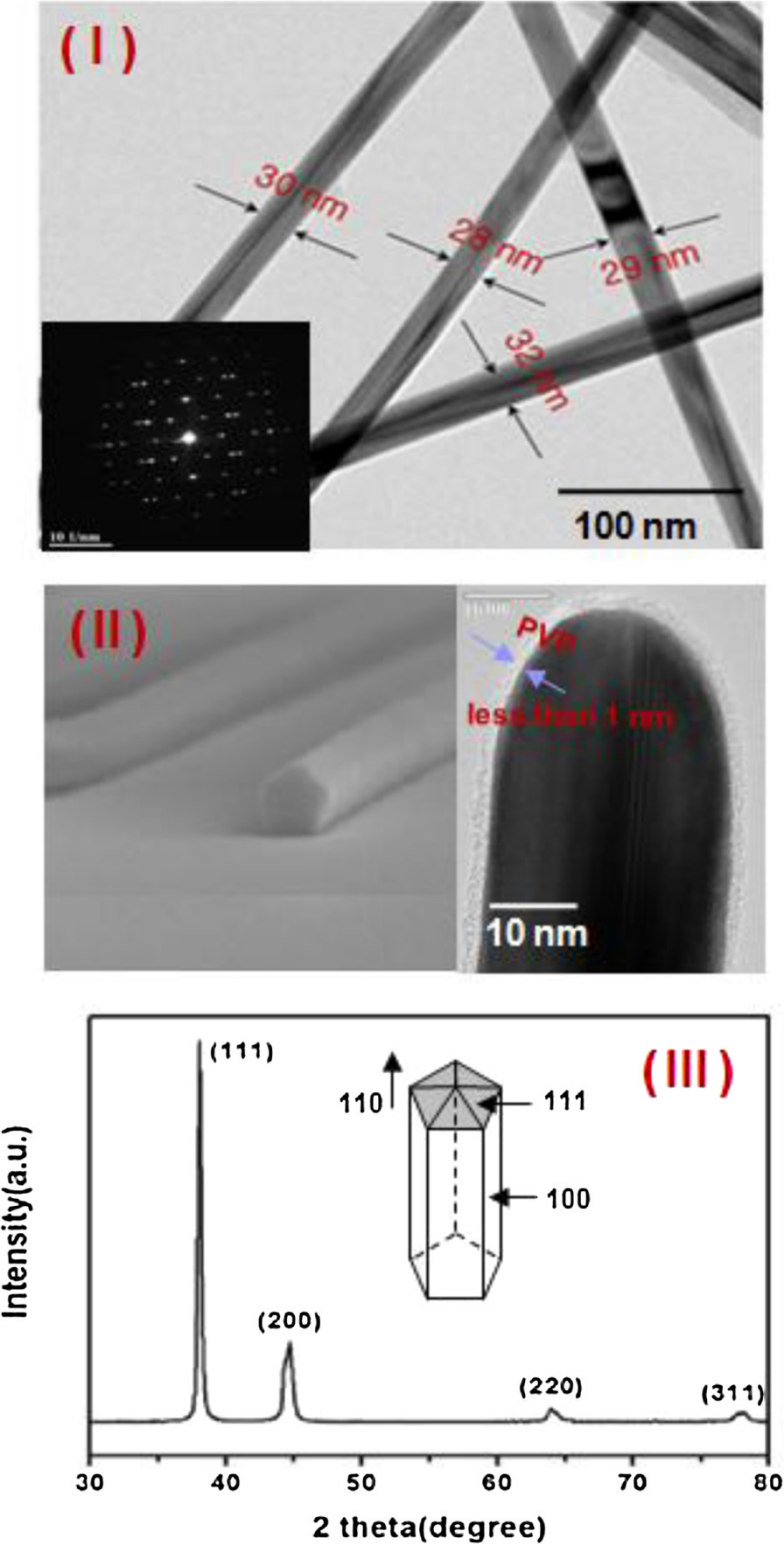
**TEM images of the Ag NWs grown in this investigation. (I)** TEM image of the synthesized Ag NWs. The inset of **(I)** displays the SAED pattern of the Ag NW with a twinned structure. **(II)** TEM image of the tip of an individual pentagonal Ag NW capped with a PVP layer less than 1 nm thick. **(III)** XRD pattern of the Ag NWs.

Figure 4 displays the TEM images of the synthesized Ag NWs. As shown in Figure 4I, the TEM images indicate that the diameter of each nanowire is uniform, with a narrow size distribution. The high-resolution TEM images provided further insight into the structure of the Ag NWs (Figure 4II), in which the NWs were determined to grow along the [110] direction. In particular, Figure 4II displays the tip of an individual Ag NW, and the contrast clearly confirms that the wire was equally divided by a twin plane parallel to the longitudinal axis. A previous study [15] has demonstrated a low threshold for the twinning parallel to the [111] face of Ag, which was determined from the grown bi-crystals twinned along the [111] plane. Additionally, uniform and extremely pure Ag NWs capped with PVP and less than 1 nm in thickness were obtained through the IL synthesis. As shown in Figure 4III, the thickness of the PVP capped on the Ag NW surface was less than 1 nm. The X-ray diffraction (XRD) pattern taken from the sample prepared in TPA indicates that the crystal structures of these nanowires were face-centered cubic (fcc) (Figure 4III). Figure 4III displays the XRD patterns of the nanowires, and it is seen that all diffraction peaks can be indexed according to the fcc phase of Ag. It is worth noting that the intensity ratio of the reflections at [111] and [200] exhibits relatively high values, indicating the preferred [111] orientation of the Ag NWs. The longitudinal axis was oriented along the [110] direction, and all Ag NW diameters were found to be in the narrow range between 28 and 33 nm, as shown in Figure 4I.

**Figure 5:**
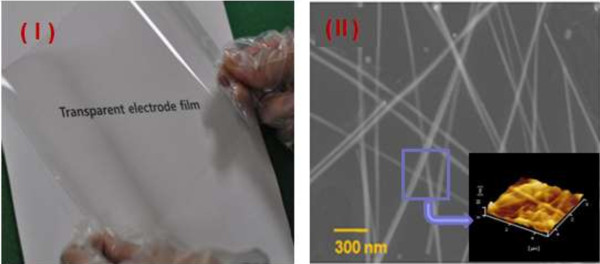
**Optical image of the Ag NW film and SEM and AFM surface morphologies. (I)** Optical image of the Ag NW film directly cast from the Ag NW solution and **(II)** SEM and AFM surface morphologies of the resulting randomly dispersed Ag NW network film.

In contrast, to observe the optical and electrical performances for transparent electrodes, pure Ag NWs synthesized by the abovementioned method were fabricated in the form of two-dimensional (2-D) films via a casting process. The synthesized Ag NWs with an average length of 50 μm and an average diameter of 30 nm (Figure 2) dispersed in H_2_O can be easily blended with a small amount of binder resins with some surfactant. This blended solution was directly deposited or cast on a plasma-treated polyethylene terephthalate (PET) substrate by a wet process coating technique such as a bar and/or spray coater for film formation (a casting film sample is shown in Figure 5). These 2-D film structures consisting of a network of approximately 30-nm-sized Ag NWs as shown in Figure 5 are expected to be sufficiently transparent, owing to the low intensity of scattered light. As a result, we could obtain highly transparent Ag NW networked films with a sheet resistance of 20 Ω/sq and transmittance of 93% (PET film-based) with a low haze value. The morphologies of the resulting randomly dispersed Ag NW networks were examined by SEM and atomic force microscopy (AFM), as shown in Figure 5I. Untangled extremely uniform and orderly NWs were observed.

**Figure 6:**
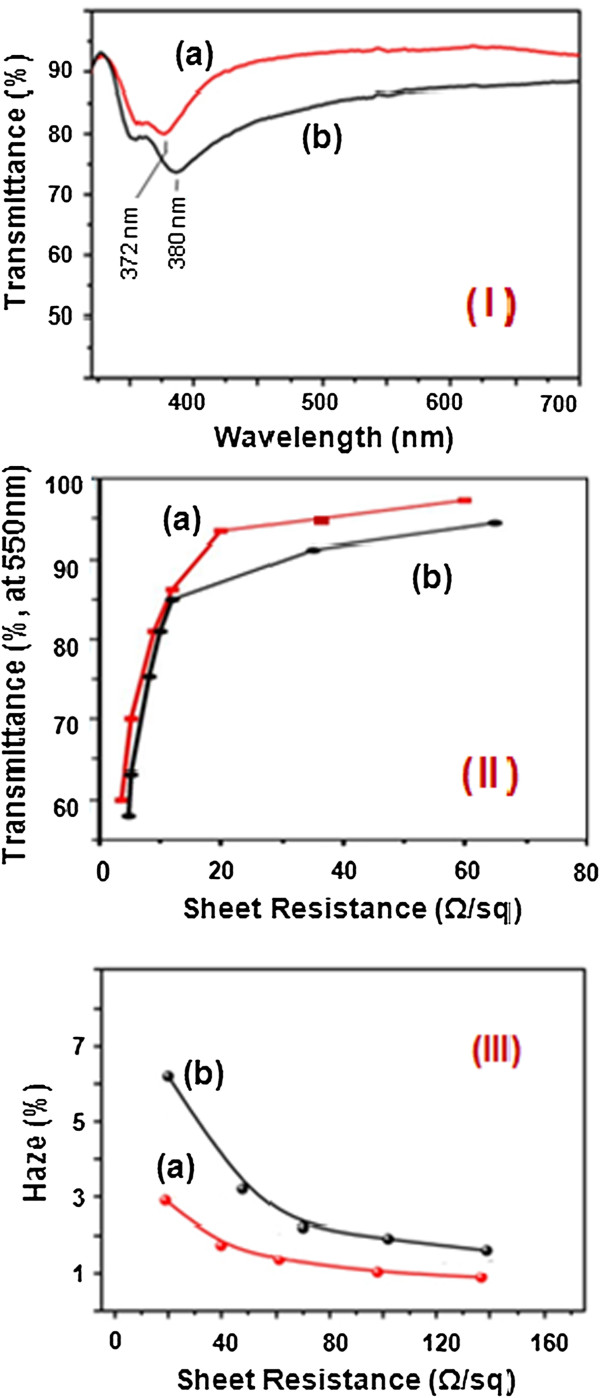
**Light transmittance spectra, changes of optical transmittance, and haze value. (I)** Light transmittance spectra of the Ag NW thin films (PET-based); transmittance values (at 550 nm) of (a) 93% for the sample containing Ag NWs of 30 ± 3 nm in diameter and (b) 86% for the sample containing Ag NWs of 45 ± 3 nm in diameter. **(II)** Changes of optical transmittance and **(III)** haze value according to the sheet resistance of the Ag NW films; (a) sample of Ag NWs of 30 ± 3 nm in diameter and (b) sample of Ag NWs of 45 ± 3 nm in diameter.

We also measured the electrical conductivity of these Ag NWs by determining the resistance of an individual nanowire at room temperature using the two-probe method. In this case, an Ag NW approximately 30 nm in diameter was aligned across two gold electrodes that had been patterned on an insulating layer of silicon oxide. The current (*I*) was measured while different DC potentials (*V*) were applied to these gold electrodes. An electrical conductivity of approximately 0.3 × 10^5^ S/cm was calculated from the linear *I*-*V* curve. Additionally, the 2-D film structures consisting of the Ag NW networks (fabricated by the abovementioned process, as shown in Figure 5) exhibited a sheet resistance as low as 20 Ω/sq with a transmittance of 93% (the sheet resistance of the Ag NW films was measured using the four-probe method). These sheet resistance value and transparency nearly match the properties of ITO films. In particular, the optical properties (transmittance and haze) in the Ag NW network structure are directly related to the diameter size of the Ag NWs. The light transmittance difference of the as-cast Ag NW films with diameters of 30 ± 3 nm and 45 ± 5 nm is shown in Figure 6I. The 2-D Ag NW film formed by a network of wires of 30 ± 3 nm in diameter was at least 3% or more transparent than the film-containing wires of 45 ± 5 nm in diameter, when both films were tested under similar sheet resistance conditions (approximately 20 Ω/sq). Furthermore, the Ag NW film-containing wires of 30 ± 3 nm in diameter consistently exhibited a lower sheet resistance than the film-containing wires that were 45 ± 5 nm in diameter with a similar transparency with respect to the film thickness or density, as shown in Figure 6II. In contrast, for the same sheet resistance value, the light transmittance of the Ag NW film of 30 ± 3 nm in diameter was at least 5% or more than that of the Ag NW film of 45 ± 3 nm in diameter. This difference of 5% transmittance is attributed to size effects. Overall, it is clear that the transmittance of the Ag NW film containing small-diameter NWs improved more than that of the film containing large-diameter NWs, due to the low intensity of scattered light. However, the 2-D Ag NW films formed by a network of NWs with a diameter of 30 ± 3 nm were sufficiently transparent comparable to ITO. In Figure 6III, the difference of haze value between Ag NW films with diameters of 30 ± 3 nm and 45 ± 5 nm is shown as a function of sheet resistance. The haze value of the 30-nm-diameter wires was at least 1% or less than that of the 45-nm diameter wires, as shown in Figure 6III. In general, the haze value is known to be directly related to the size of the Ag NWs concerned with scattered light, which directly impacts their optical properties.

## Conclusions

The present work demonstrates that thin and uniform Ag NWs can be synthesized using ILs (a mixture of TPAC and TPAB) as a soft template salt when employing the PVP-assisted polyol process. Pentagonal structures twinned along the [111] plane are subsequently produced, and the nanowire dimensions, particularly the diameters, can be controlled by the composition of the ILs. Ag can be directly grown into thin nanowires with diameters of 30 ± 3 nm and long lengths of approximately 50 μm. Additionally, the characteristic SPR of thin Ag NWs was observed at 372 nm in the absorbance spectra, which is evidence of the formation of NWs. Furthermore, these thin and long Ag NWs were determined to possess an electrical conductivity of approximately 0.3 × 10^5^ S/cm, and the sheet resistance of a 2-D percolating Ag network was found to be 20 Ω/sq with an optical transmittance of 93%. The light scattering intensity was largely reduced and thus improved the optical properties. It is obvious that these transparent conducting Ag NWs have the potential to outperform conventional ITO thin films, especially when used in flexible OLED devices as a possible electrode layer.

## Competing interests

The authors declare that they have no competing interests.

## Authors’ contributions

M-HC and H-AC participated in the experiment design, carried out the synthesis, tested the thin films, and helped draft the manuscript. Y-SK and E-JL participated in the structure analysis of the synthesized silver nanowires and fabrication of the film. J-YK wrote the manuscript and supervised the work. All authors read and approved the final manuscript.
